# Interfering With Inflammation: Heterogeneous Effects of Interferons in Graft-*Versus*-Host Disease of the Gastrointestinal Tract and Inflammatory Bowel Disease

**DOI:** 10.3389/fimmu.2021.705342

**Published:** 2021-06-24

**Authors:** Eileen Haring, Robert Zeiser, Petya Apostolova

**Affiliations:** ^1^ Department of Medicine I, Medical Center - University of Freiburg, Faculty of Medicine, University of Freiburg, Freiburg, Germany; ^2^ German Cancer Consortium (DKTK), Partner Site Freiburg and German Cancer Research Center (DKFZ), Heidelberg, Germany

**Keywords:** graft-versus-host disease, inflammatory bowel disease, interferon, intestine, ulcerative colitis, Crohn’s disease

## Abstract

The intestine can be the target of several immunologically mediated diseases, including graft-versus-host disease (GVHD) and inflammatory bowel disease (IBD). GVHD is a life-threatening complication that occurs after allogeneic hematopoietic stem cell transplantation. Involvement of the gastrointestinal tract is associated with a particularly high mortality. GVHD development starts with the recognition of allo-antigens in the recipient by the donor immune system, which elicits immune-mediated damage of otherwise healthy tissues. IBD describes a group of immunologically mediated chronic inflammatory diseases of the intestine. Several aspects, including genetic predisposition and immune dysregulation, are responsible for the development of IBD, with Crohn’s disease and ulcerative colitis being the two most common variants. GVHD and IBD share multiple key features of their onset and development, including intestinal tissue damage and loss of intestinal barrier function. A further common feature in the pathophysiology of both diseases is the involvement of cytokines such as type I and II interferons (IFNs), amongst others. IFNs are a family of protein mediators produced as a part of the inflammatory response, typically to pathogens or malignant cells. Diverse, and partially paradoxical, effects have been described for IFNs in GVHD and IBD. This review summarizes current knowledge on the role of type I, II and III IFNs, including basic concepts and controversies about their functions in the context of GVHD and IBD. In addition, therapeutic options, research developments and remaining open questions are addressed.

## Introduction

The intestine poses a unique environment for the immune system. Innate and adaptive immune cells cooperate at this physiological barrier surface to maintain homeostasis and prevent infection with pathogens that are ingested with the food. An interplay between intestinal microbiota and nutritional metabolites further shapes the microenvironment. Loss of homeostasis between these factors may result in local inflammation. Two disease groups that elicit immune-mediated intestinal tissue damage are graft-versus-host disease (GVHD) and inflammatory bowel disease (IBD). These diseases develop in distinct situations. IBD is the most prevalent autoimmune condition of the intestine, while the occurrence of GVHD is limited to the specific case of a patient who has received an allogeneic hematopoietic cell transplantation (allo-HCT). Nevertheless, both diseases share similar pathophysiological mechanisms. One of them is the involvement of interferons (IFNs) as soluble mediators shaping the microenvironment. Here, we review recent literature about the role of IFNs in intestinal GVHD and IBD. We first provide an introduction about the biology of both disease groups, followed by an overview of IFN production and signaling. In the second part, we discuss the function of different IFN subtypes in preclinical models and clinical studies of GVHD and IBD.

## Pathophysiology of Graft-*Versus*-Host Disease

Allo-HCT is one essential curative therapy option for malignant diseases of the hematopoietic system such as leukemia or lymphoma. It is also used for the treatment of benign disorders, most predominantly immunodeficiency syndromes (1). The allograft recipient is conditioned for the graft transplantation by the administration of chemotherapy, and in some cases irradiation, followed by the intravenous infusion of allogeneic hematopoietic stem cells (HSCs). Along with HSCs, the allogeneic graft contains also pre-existing mature lymphocytes (2). These donor immune cells are able to attack residing tumor cells when the allo-HCT is performed to treat a malignant underlying disease. This process is termed graft-versus-leukemia or graft-versus-tumor effect and is essential for long-term malignancy control (3). On the other hand, the donor immune cells (especially T cells) can also harm healthy tissues in the recipient. This inflammatory process is known as GVHD and its high morbidity and mortality limit the therapeutic success of allo-HCT. Classically, GVHD presents itself in two different clinical manifestations: acute GVHD (aGVHD) and chronic GVHD (cGVHD). The main target organs in aGVHD are the liver, the skin and the gastrointestinal (GI) tract. Clinical symptoms may develop within a few weeks after allo-HCT and include a maculopapular rash, hyperbilirubinemia, cholestasis as well as voluminous diarrhea, abdominal pain and bleeding (4). In addition to the affected tissues in aGVHD, any other organ system such as oral, esophageal and ocular systems, but also hair, nails, genitalia, joint fascia and lungs can be involved in cGVHD, which occurs late (in most cases, up to one year) after allo-HCT (5). GVHD is a frequent complication of allo-HCT with 30-50% of all allo-HCT recipients being affected (4). Due to its high prevalence and the diversity of involved organs, GVHD poses a major challenge in the care of allo-HCT recipients together with the risk of infections and malignancy relapse.

**Figure 1:**
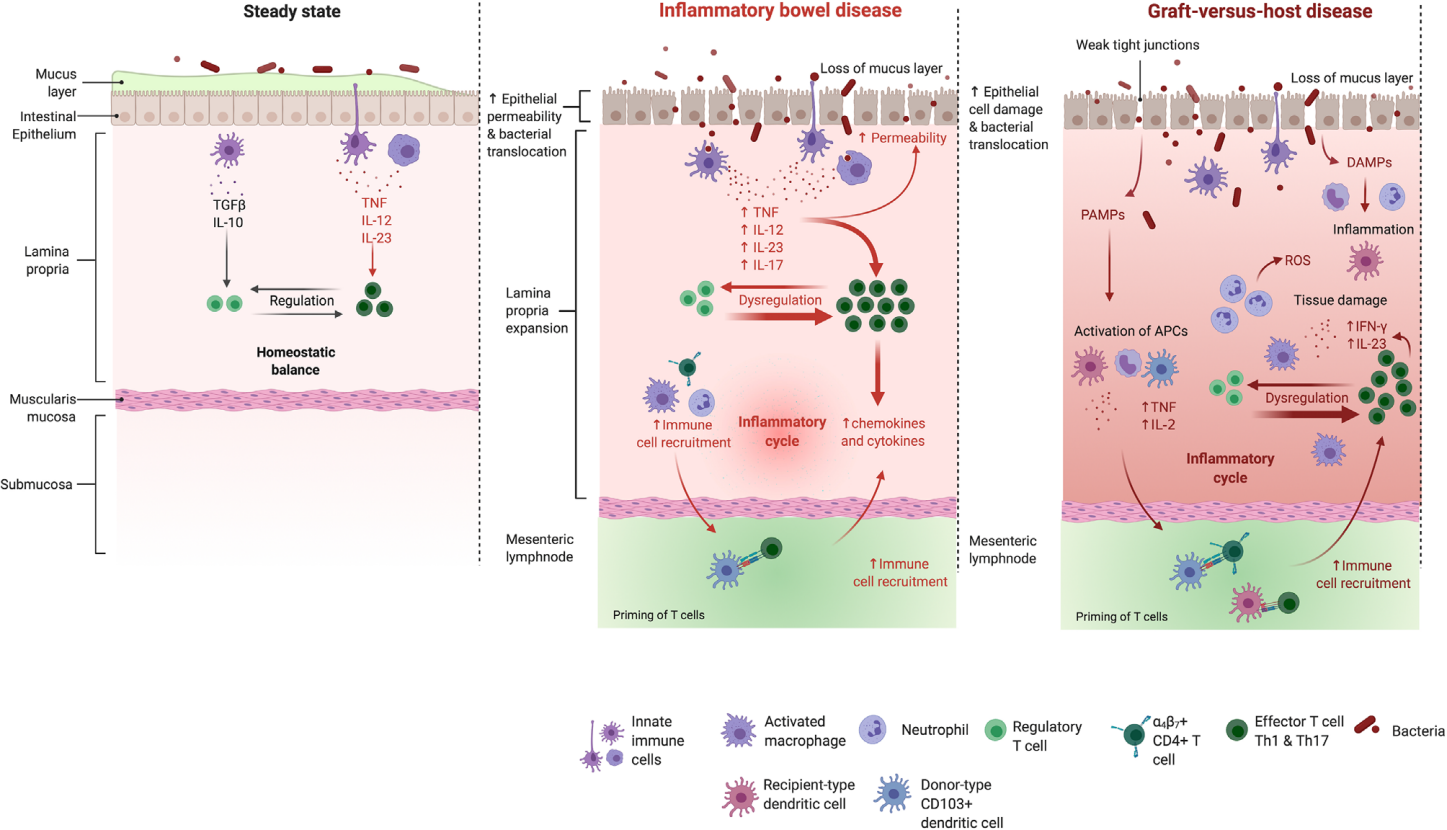
The intestinal mucosa in the healthy bowel, in IBD and GVHD. Mechanisms maintaining the healthy intestinal barrier (e.g. a thick mucus layer and tight junctions) are disrupted in the mucosa of IBD patients. The balance between effector- and regulatory T cells gets disturbed which leads to an activation of different effector T cell subtypes and their uncontrolled migration into the inflamed intestine. Also in GHVD pathogenesis, the intestinal barrier gets disrupted. Intestinal injury due to administered conditioning regiment leads to the translocation of bacteria, PAMPs and DAMPs. Neutrophils are recruited and promote tissue damage through reactive oxygen species secretion. The costimulatory activity of host antigen presenting cells is enhanced. Donor T cells are primed, proliferate and differentiate in response to host stimulatory APCs. Th1 cytokines (IFN-γ, IL-2, and TNF) and chemokines are released in large quantities. A complex cascade including cellular mediators (e.g. cytotoxic T cells and macrophages) and soluble inflammatory effectors (e.g. TNF and IFN-γ) collectively promotes local tissue damage and further drives the inflammatory cycle. IL, interleukin; TGFβ, transforming growth factor β; TNF, tumor necrosis factor; IFN, interferon; ROS, reactive oxygen species; PAMPs, pathogen associated molecular patterns; DAMPs, danger associated molecular patterns. Adapted from “Immune response in IBD”, by Biorender.com (2021). Retrieved from https://app.biorender.com/biorender-templates.

The development of GVHD is a complex interplay between hematopoietic and non-hematopoietic cells, soluble mediators, metabolites and bacteria. The key cellular mediators of GVHD are the alloreactive T cells, which are contained in the donor graft and become activated by different signals during disease development. The conditioning regimen prior to allo-HCT damages tissues of the recipient resulting in the release of both danger- and pathogen-associated molecular patterns (DAMPs and PAMPs). Together with inflammatory cytokines such as TNF and IL-6, a local inflammatory environment is established (6–8). Antigen-presenting cells (APCs) get activated and present peptides from the recipient. This in turn leads to the activation and expansion of the alloreactive T cells, which recognize the host peptides as foreign based on differences in major and minor histocompatibility antigens between donor and recipient. Cellular mediators of tissue damage in the patient comprise cytotoxic CD8^+^ T cells, NK cells as well as macrophages (7). They act together with soluble inflammatory effectors to promote local tissue destruction and further enhance inflammation (**Figure 1**). Involvement of the GI tract is associated with a high morbidity and mortality (9, 10). Intestinal epithelial cell (IEC) numbers are markedly reduced in aGVHD, and their damage leads to a loss of intestinal barrier function associated with inferior survival (11). This in turn further elevates tissue damage accompanied by bacterial transmigration and therefore strengthens the local pro-inflammatory setting during disease pathogenesis (8, 12). Besides epithelial cells, intestinal stem cells (ISCs) and Paneth cells are a major target of GVHD. ISCs are located at the bottom of the intestinal crypts where they proliferate and differentiate to regenerate all intestinal cell types. Several studies could underline that damage of the ISC is a key event in disease pathogenesis and that supporting their regeneration improves GVHD outcome (13–16). Paneth cells are located in close proximity to the ISCs. They produce antimicrobial peptides, such as lysozyme and defensins. Paneth cell number reduction in GVHD has been associated with microbial dysregulation through the reduction of intestinal α-defensins (17, 18). In humans, low Paneth cell numbers at the onset of GVHD correlated with inferior survival (19). Besides Paneth cells, L cells were recently shown to be a target of aGVHD and their loss causes a lack of the enteroendocrine hormone Glucagon-like-peptide-2 (GLP-2) (16). Another major determinant of GVHD severity is the intestinal microbiome. Multiple studies observed a loss of general bacterial diversity with a shift between beneficial and detrimental bacterial species during GVHD (20–22). Fecal microbiota transplantation has shown efficacy in patients with steroid-resistant GVHD (23–26) pointing out to the significance of microbial regulation of inflammation. Due to this complex, multi-layer pathogenesis, GVHD has proven difficult to treat in a significant number of patients.

## Pathophysiology of Inflammatory Bowel Disease

Inflammatory bowel disease (IBD) is a group of chronic and recurrent nonspecific inflammatory autoimmune diseases of the intestinal tract. Several factors including genetic predisposition, environmental factors, the intestinal microbiome as well as immune dysregulation play a role for the development of IBD (27–29). The two main clinical presentations of IBD comprise Crohn’s disease (CD), characterized by inflammation in different parts of the intestine, and ulcerative colitis (UC), which leads to persistent inflammation and ulcers limited to the colon (30, 31). CD and UC are chronic, often progressive diseases. The major clinical symptoms are chronic diarrhea, abdominal pain and bleeding, weight loss, nausea, vomiting and fatigue (32). IBD can be accompanied by a wide range of serious complications such as abscesses, fistulas and inflammation-associated colon cancer. In particular in the case of CD, extra intestinal manifestations are frequent, with skin, eyes, bones and joints being affected (33, 34).

There has been strong evidence showing that - similarly to GVHD - a loss of intestinal barrier integrity contributes to the initiation of IBD (11, 35). The barrier disruption allows translocation of microbes and microbial products which results in the engagement of pattern-recognition-receptors (PRRs) present on IECs and various hematopoietic as well as non-hematopoietic cells within the mucosa. PRR stimulation ultimately leads to the induction of an immunologic response *via* inflammasome activation and the production and release of pro-inflammatory cytokines as well as chemokines (36, 37) (**Figure 1**). Previous studies could elucidate that an imbalance between pro-inflammatory Th17 cells and anti-inflammatory regulatory T cells (Tregs) was essential in the context of IBD initiation, progress and maintenance (38–40). Proinflammatory cytokines, including TNF and IFN-γ, were shown to be key players in driving the excessive and imbalanced immune response, accompanied by harmful leukocyte infiltration and intestinal mucosal damage (41, 42). Furthermore, it was demonstrated that the microbiome played a key role in IBD onset and pathogenesis as it was seen that the development of intestinal inflammation in mice was abolished under germ-free conditions in a variety of mouse models (43). In addition to similar intestinal clinical manifestations, both GVHD and IBD also share extra intestinal organ involvement such as bile duct damage, amongst others (37). Underlining the shared aspects of disease pathologies, corticosteroids and other immunosuppressive medication is utilized in both conditions (44, 45). Newer approaches in IBD therapy suggest that the earlier utilization of advanced therapies, including immunomodulatory drugs such as thiopurines and methotrexate effectively reduces disease progression and minimizes long-term complications for the patient (46, 47).

## Interferon Production and Signaling

**Figure 2:**
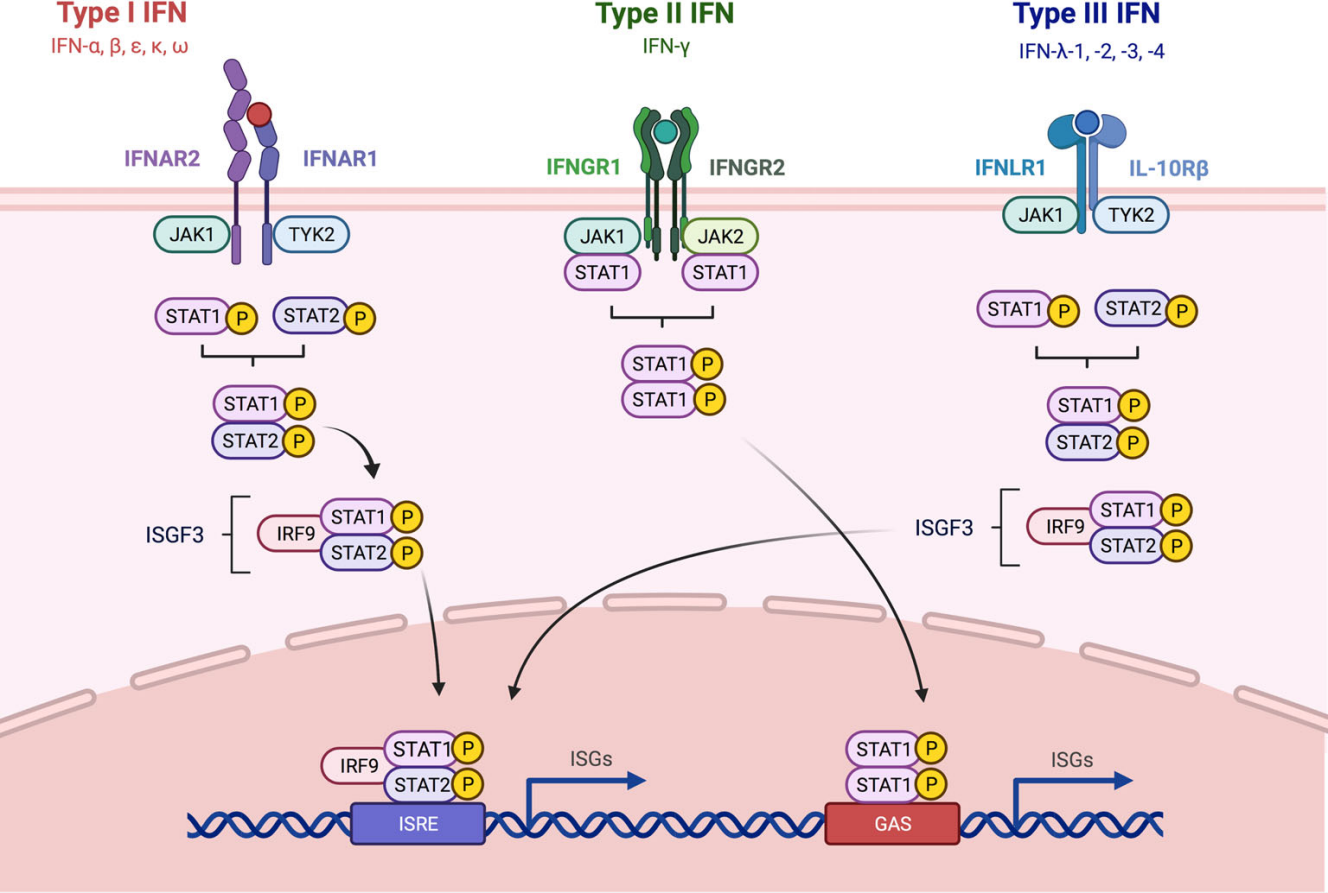
Overview about Type I, -II and -III IFN signaling pathways. The three different types of IFNs discussed in this review signal through distinct receptor complexes on the cell surface. Type I IFNs act through the type I IFN receptor which is composed of the two subunits IFNAR1 and IFNAR2; Type II IFNs act through heterodimers consisting of IFNGR1 and 2 IFNGR2 and type III IFNs signal *via* heterodimers consisting of IL-10R2 and IFNLR1. Binding of type I and type III IFN to their respective receptor complexes triggers phosphorylation of associated JAK1 and -2, leading to the recruitment and subsequent phosphorylation of STAT1 and -2. STAT 1 and -2 form together a complex, which in turn recruits IRF9 which results in the formation of ISGF3. Engagement of type II IFN to the IFNGR1/2 complex leads to phosphorylation JAK1 and -2, and subsequently STAT1 is recruited and phosphorylated. Both IRF9 and the homodimer consisting of phosphorylated STAT1 can then translocate into the nucleus and bind to ISRE and GAS elements in the promoter region of ISGs, leading to the induction of the expression of antiviral genes. IFN, interferon; STAT, signal transducer and activator of transcription; JAK, Janus kinase; TYK, tyrosine kinase; IL, interleukin; IFNAR, interferon alpha receptor; IFNGR, interferon gamma receptor; IFNLR, interferon lambda receptor; ISGF3, interferon-stimulated gene factor 3; IRF9, interferon regulatory factor 9; ISRE, interferon-stimulated response element; GAS, interferon gamma activated site; ISG, interferon-stimulated gene. Adapted from “Interferon pathay”, by Biorender.com (2021). Retrieved from https://app.biorender.com/biorender-templates.

IFNs are a group of cytokines which in humans can be divided into three categories: type I IFNs (comprising IFN-α, IFN-β, IFN-ϵ, IFN-κ, and IFN-ω), type II IFNs (IFN-γ) and type III IFNs (IFN-λ1, IFN-λ2, IFN-λ3, IFN-λ4), also known and described as IFN-like molecules. Type I IFNs bind to a common cell surface receptor named type I IFN receptor, which is composed of the two subunits IFNAR1 and IFNAR2 and is expressed on all nucleated cells (48, 49). The subunits are associated with the Janus activated kinases (JAKs) tyrosine kinase 2 (TYK2) and JAK1. Receptor engagement by type I IFN leads to tyrosine phosphorylation of signal transducer and activator of transcription 1 (STAT1) and STAT2. Together with interferon regulatory factor 9 (IRF9), both phosphorylated STAT proteins form a complex which is known as IFN-stimulated (IFN-stimulated gene (ISG) factor 3) ISGF3 complex (50, 51). This complex translocates into the nucleus and binds to IFN-stimulated response elements (ISREs) to initiate the transcription of different ISGs which mediate various biological processes (52). Aside from STAT1 and -2, type IFN I signaling can also induce STAT3-6, so that various homo- and heterodimer combinations can assemble (53). In contrast to the downstream signaling of the ISGF3 complex, which is comprised of STAT1, -2 and IRF9, the other complexes bind to another type of regulatory element: the IFN-γ-activated site (GAS) element. Various ISGs contain either only ISREs or GAS elements in their promoter regions, whereas some contain both. This shows that type I IFN signaling can induce a variety of functionally distinct target genes, although the exact mechanism behind the regulation of the various STAT engagements is not fully understood yet (51). IFN-γ, as the only type II IFN, binds to a different cell surface receptor: the type II receptor, composed of the two subunits IFNGR1 and IFNGR2, which are associated with JAK1 and JAK2, respectively (49, 54). Here, the STAT1 homodimer is the essential transcription factor, which gets activated *via* phosphorylation. Since the STAT1 homodimer does not bind to IRF9, it is not able to bind ISREs. Therefore, type II IFN signaling only induces transcription of genes, which possess GAS elements in their regulatory regions (55–57). Finally, all type III IFNs bind to a receptor complex composed of two subunits: CRF2-12 (also designated as IFN-λR1) and CRF2-4 (also known as IL-10R2), together named 65R1. Type III INFs are the “youngest” group of IFNs and were only discovered in 2003 (58, 59). Similar to type I IFNs, signaling *via* type III IFNs induces the trimerization of the heterodimer STAT1-STAT2 with IRF9 resulting in the assembly of the ISGF3 complex. Type III IFN signaling can therefore activate ISG with ISREs or GAS elements in their regulatory region (60) (**Figure 2**). In contrast to the wide receptor expression for type I and II interferons, expression of type III interferon receptor seems to be limited to certain tissues and cell types. Keratinocytes and epithelial cells of the lung and the GI tract have been shown to express significant amounts of IFNLR1. Interestingly, so far plasmacytoid dendritic cells (pDCs) seem to be the only hematopoietic cell type which is responsive to type III IFNs (61, 62). The various impacts and functions of ISGs were recently covered in a comprehensive review by Schoggins (63).

## Immunoregulatory Effects of IFNs

Type I IFNs have a wide range of functions and are produced by various cell types in response to pathogenic - mostly viral but also bacterial - infections. The functions include anti-pathogen activity as well as anti-proliferative actions. During the last decades it became also clear, that type I IFN can exert immunomodulatory actions on cells of both the innate and the adaptive immune system (54, 64). Type I IFN production is triggered by various PRRs including Toll-like receptors (TLRs), RIG-I-like receptors (RLRs) as well as NOD-like receptors (NLRs), that can be activated by sensing viral nucleic acids and other stimuli. PRR activation leads to the rapid induction of type I IFN during the early phases of viral infections before the adaptive immune response including antiviral CD8^+^ T cells is induced and established (65). As part of the innate immune system, plasmacytoid dendritic cells (pDCs) were implied as the most predominant IFN-α producing cells (66–68). Type I IFNs indirectly affect T cell activation by inducing the maturation, migration and antigen presentation capacity of DCs to facilitate their adaptive antiviral immune response (69–74).

Natural killer (NK) cells, natural killer T cells (NKT), CD4^+^ T helper type 1 (Th1) cells, CD8^+^ cytotoxic T cells as well as γδ T cells are the main IFN-γ-producing cell types (75). IFN-γ plays an essential role in MHC class I and II antigen presentation pathways. It induces the upregulation of MHC class I cell surface expression which is important for the immune response against intracellular pathogens and essential for the actions of cytotoxic effects of CD8^+^ T cells. All exact impacts of IFN-γ on genes which are associated with the MHC class I antigen presentation pathway have been reviewed by Schroder and colleagues (57). Notably, IFN-γ is the sole IFN, which is able to induce MHC class II expression on professional APCs such as DCs, macrophages and B cells. It thus plays an exclusive role in the activation of CD4^+^ T cells *via* specific MHC class II/peptide recognition (75). During the adaptive immune response, CD4^+^ Th1 cells as well as CD8^+^ cells are able to secrete IFN-γ after being activated and differentiated (63). Furthermore, IFN-γ can have both immune-stimulatory as well as -suppressive roles in all stages of the tumor immunoediting process (76–78).

Type III IFNs can promote an antiviral response, which is similar to the response to type I IFNs (79). A distinct feature of both IFN types lies in the production of the respective cytokine and the distribution of the corresponding receptors. Type III IFNs are especially important at epithelial barrier surfaces. Epithelial cells of the respiratory but also intestinal tract express high amounts of IFNLR1 demonstrating a predominant role of type III IFNs in the epithelial antiviral host defense (62, 80, 81).

## Role of IFNs in the Murine and Human Intestine

IECs play a key role in balancing the intestinal immune homeostasis. They need to act tolerogenic to the vast amount of bacterial commensals but at the same time also be responsive to detrimental pathogens. In this context, there is increasing evidence that both type I and type III IFNs are important for the maintenance of the intestinal epithelial barrier integrity and the control of adaptive immune responses including antiviral responses (81, 82). In the intestine, type I IFNs are for example continuously produced by CD11c^+^ DCs of the lamina propria (83). In contrast to that, it was shown, that murine IECs preferentially expressed type III IFNs over type I IFNs upon infection with human reoviruses and that they expressed higher levels of IFNLR1 than IFNAR1 and -2 compared to the underlying lamina propria (84, 85). This differential distribution of IFN receptors demonstrated that type III IFN IFN-λ could be seen as the very first line of defense against intestinal pathogens and might represent a nonredundant part of the innate antiviral immune response (81). Proof for that concept was established by studies, which revealed that IFN-λ signaling in IECs was protective against intestinal virus infection using mice with a conditional knock-out of IFNRL1 in the intestine. Depleting IFN-λ signaling in IECs led to an increase in intestinal virus replication and fecal shedding (86). Additionally, it was demonstrated, that administration of IFN-λ could cure intestinal virus persistence of norovirus even independent of the adaptive immune system (87). Though IFNs type III were shown to have this very essential role for the antiviral response of IECs, type I IFNs are not expendable. The same studies underlined the hypothesis, that type I IFNs, rather than protecting the IECs directly, were in fact essential for the prevention of a systemic spread of the intestinal viral infection (85, 87). Broggi and colleagues concluded, that in the intestine, type I and III IFNs acted together in a compartmentalized system. In this synergy, the type III IFN IFN-λ had the primary role in protecting the epithelial barrier, and type I IFNs only came into action once this barrier has been penetrated by invading pathogens (88).

Nevertheless, IFN-α was found to prevent staurosporine-induced apoptosis murine model of the developing intestine *via* induction of the GTPase guanylate-binding protein-1 (GBP-1) expression, which was involved in regulating intestinal barrier function (89, 90). Using mice deficient for IFNAR1, it was demonstrated, that type I IFN signaling could determine Paneth- and goblet cell numbers in the intestine. Both epithelial cell hyper-proliferation and increased tumor burden were associated with the IFNAR1-deficient intestinal epithelium in a colitis-associated cancer model. Interestingly, intestinal cell hyper-proliferation as well as tumor promotion were reversed in the IFNAR1-deficient mice upon co-housing with WT littermates, underlining that IFNAR1 in IECs contributed to the regulation of the host-microbiome relationship which had consequences for intestinal cell regeneration as well as tumor formation (89). In the human setting it could also be demonstrated, that intestinal virus infection preferentially induced the upregulation of type III IFN to a higher extent compared to type I IFN, leading to a protective effect of type III IFN on the IECs expressing type III IFN receptors (91, 92). Recently it was discovered that, similar to the murine system, IFN-λ played an essential role in the context of epithelial cell protection during intestinal virus infection in humans. Human intestinal epithelial cells lacking IFNLR1, but not those lacking IFNAR1, showed diminished ability to control SARS-CoV-2 infection and replication in the intestine (93). Altogether, studies in both murine and human setting suggest a model, in which IECs favor type III IFN-mediated signaling over type I IFN signaling upon viral infection. This model allows an effective innate response to virus infection without triggering a systemic inflammatory process *via* type I IFN production and -signaling, thereby maintaining local intestinal gut homeostasis (91). In contrast to the protective role of type III IFNs on IECs, type II IFN IFN-γ was found to have negative effects on IECs and intestinal homeostasis (94–96). It was demonstrated, that IFN-γ produced by immune cells during mucosal immune response has destructive effects on Paneth cells (97, 98).

Two important regulators of IFN production are intestinal microbiota and their metabolites. Depletion of intestinal bacteria by antibiotic treatment reduced type I interferon responses in chicken after a challenge with influenza virus (99). In mice undergoing influenza A infection, decontamination of the gut by administration of antibiotic-containing water decreased ISG expression in stromal cells of the lung, indicating that changes of the intestinal microbiome have an impact on interferon signaling in the whole body. Interestingly, fecal transplantation was able to reverse the effects of antibiotic treatment and restored ISG expression (100). In a recent study, mice undergoing oral antibiotic treatment were also more susceptible to Chikungunya virus infection. The authors found by single-cell RNA sequencing that antibiotic treatment reduced type I IFN production by pDCs and subsequent expression of ISGs in infected monocytes. They further discovered that *Clostridium scingens*, by converting a primary bile acid into the secondary bile acid deoxycholic acid, was able to reconstitute IFN production by pDCs (101, 102). Other metabolites produced by intestinal microbiota, such as short-chain fatty acids (SCFAs), also play a significant role in colonic homeostasis and inflammation (103, 104). SCFAs can show modulatory effects on intestinal epithelial cells and neutrophils, as well as monocytes and macrophages (105). One of the most important SCFAs is butyrate, which is produced by Clostridia and Firmicutes, among others. Early on, butyrate enema therapy was found to be able to stimulate mucosal repair in experimental models of colitis in rats (106). Accordingly, several studies have been conducted highlighting the potential beneficial effect of butyrate on the course of UC in patients (107–109). In Crohn’s disease, butyrate was administered orally to patients in the form of tablets. Butyrate is able to antagonize colonic inflammation (110) and has been found to reduce the production of pro-inflammatory cytokines including IFN-γ. It does this by acting as a histone deacetylase inhibitor and interfering with transcription of IFN-γ by inhibiting IFN-γ-induced tyrosine and serine phosphorylation of STAT1 (111–113). In 2019, Chen and colleagues investigated whether butyrate treatment could regulate the differentiation of T cells into Th1 and Th17 cell fates. They found that, on the one hand, promotion of both T cell subtypes was induced and differentially regulated (including promotion of IFN-γ expression in Th1 cell development), but most interestingly, expression of anti-inflammatory IL-10 was induced in both cases. Rag1-deficient mice receiving these butyrate-treated T cells showed less severe colitis compared with animals receiving untreated T cells. These data provide important details about how butyrate might be used therapeutically in IBD (114). Another interesting study from the same year examined the relationship between the microbiome, their intestinal metabloites, and interferons. Zhai and colleagues tested the ability of strains of Akkermansia muciniphila, which may exert probiotic effects in obesity and diabetes, to decrease inflammation in chronic colitis in mice. Both strains used (namely 139 and ATCC) were able to improve colonic inflammation when introduced into mice suffering from DSS-induced colitis. In addition, the levels of proinflammatory TNF as well as IFN-γ were reduced in the colon of the mice. Most importantly, they found that strain ATCC was able to induce the production of beneficial SCFAs (115). Also beyond intestinal inflammation, butyrate production by *Lachnospiraceae* was found to inhibit STING-activated type I IFN production by DCs (116). Conversely, beneficial lactic acid bacteria were shown to induce interferon type I secretion (117). Oral administration of the SCFA acetate mediated an IFN-β response by increasing ISG expression (118). These data suggest that intestinal bacteria and their metabolites have the capability to modulate interferon production and thus impact the innate immune response.

## Role of IFNs in GVHD

One of the very first reports about IFNs in the context of GVHD was delivered in 1987, where Reyes and Klimpel measured the production of IFN-α/β/γ in sera of mice which were lethally irradiated and subjected to allo-BMT. They found that higher IFN activity positively correlated with GVHD occurrence. With these observations, they paved the way for following research work regarding the influence of IFNs on GVHD development (119).

### Type I IFNs

There are controversial reports about the role of type I IFNs in GVHD. In early clinical studies from the 1990s, pre-transplant exogenous type I IFN administration in humans resulted in increased GVHD occurrence and transplant-related mortality (120, 121). In contrast, several experimental studies could demonstrate, that type I IFN signaling was able to positively modulate murine GVHD outcome (121–125). In 2011, Robb and colleagues were amongst the first researchers to investigate the role of type I IFNs in GVHD and GVL. Using IFNAR1-deficient mice as recipients or donors in a murine GVHD model as well as exogenous administration of IFN-α, they found that type I IFN signaling had pleiotropic effects. These included the suppression of CD4^+^ T cell-dependent GVHD and at the same time a paradoxical increase in CD8^+^ T cell-mediated GVHD (122). In 2017, Fischer and colleagues elegantly showed that mice deficient for mitochondrial antiviral-signaling protein (MAVS) or stimulator of interferon genes (STING), which are innate types of PRRs that induce the expression of type I IFNs, developed worse GVHD after allo-HCT. In line with that, they could ameliorate disease outcome triggering either the RIG-I/MAVS- or the STING pathway to induce protective type I IFN signaling and maintain intestinal epithelial barrier integrity (121, 123). Consistently, several studies could demonstrate, that the administration of type I IFN or type I IFN-inducing agonists was potent in protecting mice from GVHD in a MHC-mismatched model, when given before allo-HCT (124, 125). Interestingly, intestinal microbes that produce indole and indole derivatives, mitigate GVHD development, partly by induction of IFN type I-stimulated genes (124).

Another study investigated the synergy between IL-22, known to be a key player in promoting aGVHD development, and type I IFN (126). For this, the authors used IFNAR- as well as IL-22-deficient mice as recipients of allogeneic wild-type BM cells in combination with allogeneic T cells from either IFNAR- or IL-22-deficient donors. They observed lower GVHD severity in IFNAR-deficient recipient animals when IL-22-deficient donor T cells were transferred in a major MHC mismatch model. Therefore, interference with IL-22 and type I IFN signaling could be a novel treatment approach. Additionally, the authors could connect the increased GVHD severity to elevated STAT1 activation and CXCL10 expression. It was speculated, that the synergy between donor-derived IL-22 and recipient type I IFN signaling could favor the loss of intestinal barrier integrity in aGVHD pathogenesis (126). Also in a model of systemic sclerosis (Ssc) -like cutaneous GVHD, protection was achieved by blocking type I IFN signaling *via* usage of a neutralizing Ab against IFNAR1. Notably, the central question in this study was to elucidate the role of type I IFN blocking in SSc, and the cutaneous model of GVHD was only used to mimic this disease. The authors investigated fibrogenesis, but important features such as survival rate after GVHD induction and histopathological score of the intestine were not obtained (127). Altogether, these data show that type I IFNs signaling has complex and partly opposite effects on GVHD development, depending on the preclinical model used.

### Type II IFNs - Role of IFN-γ in GVHD

Over the last decades it became clear, that IFN-γ has pleiotropic effects in GVHD pathogenesis as well, depending on the examined cell type. It is well established, that intestinal damage during GVHD results in large parts from the increased release of IFN-γ and IL-12 from alloreactive Th1 T cells (128). IFN-γ induced intestinal cell apoptosis and, together with LPS originating from transmigrated bacteria, it stimulated the secretion of proinflammatory cytokines such as TNF, further supporting the inflammatory setting (129, 130). In mouse intestinal organoids, activated T cells induced tissue damage and reduction in Paneth cell and ISC numbers *via* IFN-γ signaling (131). Organoids deficient for the IFN-γ receptor remained unaffected by T cells, and *in vivo* IFN-γ administration elicited enteric inflammation (131). These data were supported by murine *in vivo* studies, where IFN-γ was described as the major mediator of ISC reduction in the colonic crypts (132). When GVHD was induced by T cells lacking IFN-γ or in mice deficient for the IFN-γ receptor in ISC, the stem cell compartment was protected (132). Collectively, these data indicate that IFN-γ has detrimental effects on the intestinal epithelium. In line with this hypothesis, already in 1989, Mowat described positive effects of the administration of an anti-IFN-γ antibody in two murine GVHD models (133).

Contrarily, a number of studies have also reported protective roles of the type II IFN in the context of GVHD. In a murine model of fully MHC-mismatched allo-BMT, IFN-γ-deficient donor CD8^+^ T cells, but not WT donor cells, were able to induce lethal GVHD (134, 135). GVHD protection appeared to be mediated by effects of IFN-γ on T cells, either through a direct mechanism or *via* modulation of IL-12 signaling. IL-12 is essential in promoting the differentiation of naïve T cells into Th1 cells (136). IL-12 is produced by APCs and stimulates IFN-γ production by T cells as well as NK cells (137). In lethally irradiated mice, one single injection of recombinant murine IL-12 simultaneously with the BMT led to the protection of mice against aGVHD in both in fully MHC- as well as minor antigen-mismatched strain combinations (138–140). In another study, the authors pinpointed that dose as well as timing of recombinant IL-12 administration determined whether this cytokine had protective or rather detrimental effects. They found that administration of IL-12 1-12h prior to BMT resulted in protective actions of IL-12 whereas administration more than 36h after BMT completely abrogated these positive effects (141). Interestingly, in the study of Yang and colleagues from 1999, protection against GVHD was completely lost upon treatment with the neutralizing anti-IFN-γ monoclonal antibody (mAb) R4-6A2 (141). Altogether, this led to the assumption, that IFN-γ is required for the protective effects of IL-12, but is not per se responsible for GVHD induction (142). To decipher, whether recipient or donor IFN-γ was responsible for the protective effects *via* IL-12, Dey and colleagues transplanted C57/BL6 mice with allogeneic HSCs from IFN-γ KO BALB/c mice and could not achieve prolonged survival rates *via* treatment with IL-12. This data supported the hypothesis that the IFN-γ which was mediating the protective effects of IL-12 was donor-derived. Mechanistically, the authors could show that Fas-mediated donor CD4^+^ T cell apoptosis was one of the underlying mechanisms involved in the protective effects of IL-12 on GHVD pathogenesis (139). Apart from regulation of IL-12 signaling, a direct protective role of IFN-γ was also observed using IFN-γ KO mice. In one study, the authors could show that the dosing of conditioning regimen plays a pivotal role considering disease outcome: IFN-γ KO animals were used as donors in lethal and sublethal allogeneic BMT experiment using total body irradiation TBI as conditioning. For recipients of lethal doses of TBI, loss of donor IFN-γ was detrimental whereas recipient of sublethal doses, the loss of IFN-γ was protective (143). Consecutive studies showed that IFN-γ deficient CD8^+^ T cells induce more severe GVHD in models with major and minor histocompatibility mismatch (134). These results were presumably based on the loss of apoptosis induced in activated CD8^+^ T cells by IFN-γ. In line with these findings, another study could prove, that the IFN-γ receptor signaling was the major pathway responsible for the migration of both conventional- but also regulatory T cells to GVHD target organs. Altered trafficking of both T cell types was mediated by expression of CXCR3 which was connected to IFN-γ receptor signaling (144). Collectively, these reports provide evidence that IFN-γ regulates the alloreactive T cell pool and can prevent excessive T cell expansion.

The role of IFN-γ in intestinal GVHD remains controversial. Multiple studies observed that IFN-γ damages intestinal epithelial cells by inducing apoptosis and production of pro-inflammatory cytokines in the intestine. On the other hand, intact IFN-γ signaling appears important for the control of alloreacitve T cell expansion, differentiation and migration. Exploring which downstream cascades are responsible for the one or the other effect might open new avenues for targeted treatment.

### Type III IFNs

Type III IFNs have only recently been discovered and therefore knowledge of their role in intestinal homeostasis and inflammation is just emerging. Epithelial cells of mucosal tissues, such as the IECs, are a major target of these type of interferons (62). Both human and murine IECs show a high responsiveness to treatment with type III IFNs. Recently, mice deficient for the IFN type III receptor (IL-28 receptor alpha subunit, IL-28Rα) showed comparable thymic regeneration potential and GVHD development as wildtype mice (145). In line with these data, IL-28A protein administration did not support recovery from irradiation-induced thymus damage (145). Nevertheless, single nucleotide polymorphisms in the IFNL4 gene in donors was associated with increased risk of non-relapse mortality in humans (146). Further studies are warranted to assess the relevance of type III IFNs in GVHD.

### Modulation of IFN Signaling as a Treatment Approach in GVHD

Given the pleiotropic effects of IFNs on different cell populations involved in GVHD, it has been a challenge to develop successful clinical strategies by direct modulation of the interactions between IFN and their receptors. One indirect approach targeting IFN signaling amongst others, is the inhibition of JAK/STAT-signaling. Pre-clinical models showed, that incidence and severity of GVHD were reduced when administrating ruxolitinib, a selective inhibitor of JAK1 and -2, both being involved in the IFN-γ signaling pathway (147–149). Based on those findings, clinical trials on the potential of ruxolitinib for the treatment of glucocorticoid-refractory aGVHD showed great success and led to the approval of ruxolitinib for this indication by the Food and Drug Administration (150, 151). Another potential avenue for the use of IFN in the treatment of GVHD is related to the generation of mesenchymal stem cells (MSCs), a cell population with immunosuppressive properties. The role of IFN-γ activating MSCs has previously been described *in vitro* (152, 153). A first pilot study in patients suffering from severe steroid-resistant aGVHD could demonstrate MSCs as a promising treatment option (154). Nevertheless, development of a MSCs-based therapy for GVHD was impeded by factors such as a lack of standard protocol for the production of MSCs and the overall heterogeneity of MSCs derived from various donors and tissues (155–158). Regarding the role of IFN-γ in activating MSCs, it could be demonstrated, that MSCs primed with IFN-γ were able to reduce GVHD in NOD-SCID mice and to ameliorate survival rates when compared to animals receiving non-primed MSCs. The authors showed, that this effect was based on an induction of indoleamine 2,3-dioxygenase (IDO) *via* the IFNγ-JAK-STAT1 pathway in the MSCs, thereby enhancing their immunosuppressive properties (159). The exact mechanisms of how the various IFNs discussed in this review act in the context of GVHD remain largely unclear. It is essential to distinguish between the effects of IFNs on the hematopoietic cells of the recipient and of the donor, respectively. Furthermore, effects on the target tissues in the recipient need to be considered. Further studies are needed to elucidate the roles of IFNs in both GVHD and GVL processes after allo-HCT and to possibly make use of protective IFN administration.

## Role of IFNs in IBD

### Type I IFNs

In the context of genome-wide association studies, several genetic susceptibility loci for UC, CD or both were identified. These included genes which are essential key players in immunity and barrier function, amongst others. Several of those identified IBD-associated genes are involved in the type I IFN signaling pathway, for example the single nucleotide polymorphism (SNP) rs2284553, which affects the IFNAR1 gene. Other SNPs were found in the genes encoding JAK2 (rs10758669), TYK2 (rs11879191), STAT1 (rs1517352) and STAT3 (rs12942547), playing a role in several signaling pathways downstream of type I and III IFNs (28, 47, 160). Therefore, aberrations in the type I signaling network could promote an imbalanced immune response leading to induction of IBD (126). Appendicitis-appendectomy (AA) has been shown to reduce or prevent UC in adulthood, which was described in several clinical studies (161–164), and reviewed by Koutroubakis and colleagues (165). Similar observations were made regarding the prevention and ability to decrease CD severity (161, 163, 166). Cheluvappa and colleagues developed a model of AA to identify novel therapy options for colitis amelioration. In this model, mice undergoing AA were protected from experimental colitis in and age-, bacteria- and antigen- dependent manner. They found that AA led to dampened Th17 cell activity and autophagy, but most interestingly, that AA was driving the modulation of IFN-associated molecules. Significant upregulation of the ISGs IFIT1, IFIT2 and IFIT3 in the distal colon 28 days after AA could be measured. These genes are induced by IFNs, virus infections and PAMPs, mediating immunomodulatory and antiproliferative functions as well as apoptosis induction (167–169). The authors assigned the beneficial effects of AA to this mode (170). Similar results were obtained in a study where imiquimod, a virostatic agent, induced type I IFN expression in the mucosa of the GI and was able to protect against DSS-induced colitis. Notably, no systemic IFN response could be measured. Based on their findings, the authors suggested imiquimod as a potential therapeutic approach for IBD patients (171). Other studies implied that type I IFNs rather played a dual role in the context of intestinal inflammation and recovery from colitis (172). Protective actions could be seen in a study where DCs, when stimulated with TLR9 agonists, produced type I IFNs leading to the protection against experimental colitis in RAG1-deficient mice. Consistently, administration of recombinant IFN-β led to similar protection (173). In a follow-up study, the authors could show more in detail, that the type I IFN produced by DCs was able to inhibit colonic inflammation *via* regulation of neutrophil and monocyte trafficking into the inflamed colon (174). In a T cell-induced colitis model, the protective effect of type I IFNs was attributed to its positive influence on Tregs *via* increasing their cell numbers and the maintenance of Foxp3 expression (175, 176). In contrast to that, it was seen that the local delivery of IFN-β *via* Lactobacillus into the intestine led to an exacerbation of DSS-induced colitis accompanied by increased levels of pro-inflammatory cytokines and lower numbers of Tregs in the small intestine of mice (177). It is important to underline, that the source of IFN-β in this study was a bacterial vehicle which might have diverse and different physiological effects compared to administration of pure recombinant type I IFN. Altogether, most studies suggest that type I IFNs are protective in different preclinical models of colitis.

### Type II IFN

The type II IFN IFN-γ is one of the most highly upregulated cytokines found in IBD patients and in murine models of intestinal inflammation (41, 42, 178–180). It was demonstrated, that one aspect of the pathophysiological role of IFN-γ in IBD lied in its direct effects on the intestinal epithelium by influencing the homeostasis between cell proliferation and apoptosis *via* the regulation of converging of β-catenin signaling pathways. In the same study, it was observed, that TNF even increased the effects of IFN-γ, underlining a synergism between those two cytokines in the setting of intestinal inflammation (94).

Apart from that, several studies could show that IFN-γ also had significant effects on the intestinal vasculature. *In vitro*, it showed an overall antiangiogenic effect, including inhibition of proliferation, invasion and tube formation of endothelial cells *via* induction of the large GTPase guanylate binding protein‐1 (GBP‐1) (181–183). Based on these findings, Naschberger and colleagues could attribute GBP-1, resulting from IFN-γ upregulation in colorectal carcinoma (CRC), to an IFN-γ-dominated Th1-like immune reaction possessing potential angiostatic/antiangiogenic activity. They underlined that the microenvironment in GBP‐1‐positive CRC is dominated by IFN-γ, which was associated with an improved prognosis for the CRC patients (184). Interestingly, by using a neutralizing anti-IFN-γ antibody in a murine DSS-induced colitis model, it was shown, that IFN-γ exhibited an endogenous angiostatic activity in IBD and contributed to increased vascular permeability (179). In contrast to that, it was recently shown, that IFN-γ acted pathogenic in IBD by negatively impacting the vascular barrier by disruption of VE-cadherin, an adherent junction protein. By using endothelial cell-specific IFN-γ-receptor-KO mouse models, the authors of the study could show, that an endothelial-specific inhibition of the IFN-γ response led to an ameliorated outcome in DSS-induced colitis. Furthermore, IBD-associated vascular barrier dysfunction was also confirmed in human patients (185). Altogether and similar as in GVHD, IFN-γ remains a pleotropic cytokine with controversial roles in IBD pathology.

### Type III IFNs

Since type III IFNs are emerging as a cytokine group with specific role on epithelial barrier surfaces, several studies tested their potential role in IBD models. First data demonstrated, that IFN-λ played a protective role in a murine model of DSS-induced colitis, thereby proposing it as an anticolitogenic cytokine (81, 126). In contrast, it was found, that levels of IFN-λ were increased in inflamed ileal tissues and sera of CD patients. This was accompanied by a loss Paneth cells. Based on those findings, the authors of this study suggested, that blocking IFN-λ or reducing its concentrations in affected patients might positively affect disease outcome (186). Further studies are required to explore the therapeutic potential of IFN-λ signaling.

### Modulation of IFN Signaling as a Treatment for IBD

Studies investigating the effects of systemic administration of type IFNs to ameliorate IBD have produced controversial results. Administration of IFNs was shown to not have positive effects in the context of UC treatment (187). Overall, a Cochrane systematic literature review from 2008 investigating the efficacy and safety of type I IFN therapy (including IFN-β-1a, IFN-β-1b, IFN-α-2a, IFN-α-2b and associated PEGylated formulations) in UC showed no difference between groups of patients which were treated with type I IFNs or placebo in regards to remission achievement or symptom improvement. The authors conclude, that the data from those clinical trials do not support the use of type I IFNs to induce remission status in active UC. In accordance to the current scientific knowledge, no statistically significant benefit regarding disease amelioration could been observed in using type I IFN for the treatment of IBDs (188).

Fontolizumab, a humanized anti-IFN-γ antibody, could not induce strong clinical responses in a phase 2, randomized, double-blind, placebo-controlled, multiple-dose study in patients suffering from moderate to severe CD. Though well tolerated, administration only led to a significant decrease in C-reactive protein levels (189). The clinical development and further investigations on Fontolizumab in the context of IBD were stopped. Also eldelumab, an anti-INF-γ-inducible protein-10 (IP-10) monoclonal antibody, could not achieve the primary endpoint in a study in patients suffering from UC (190). Interestingly, when compared to other (auto-) immune related diseases such as rheumatoid arthritis or psoriasis, it becomes apparent, that in IBD, mainly TNF antagonizing monoclonal antibodies (mAbs), including infliximab, adalimumab and golimumab, show a beneficial effect (191). IFN signaling is mediated *via* intracellular JAKs and TYK2. It is therefore evident, that blocking these kinases could be a promising approach to cope with the elevated signaling of proinflammatory cytokines with proposed roles in mucosal immune cells in intestinal inflammation. Examples include the successful use of tofacitinib, blocking JAK3 activation and signaling *via* common γ-chain containing cytokines (IL-2,-4,-7,-9,-15 and -21) in CD and UC, and the selective JAK1 inhibitor filgotinib for Crohn’s disease (192–194). This indicates that JAK inhibitors might be promising approaches for clinical therapy of IBD patients.

Regarding the therapeutic use of type III IFNs, some promising first data were collected in clinical trials for the treatment of chronic hepatitis with PEGylated forms of IFN-λ (ClinicalTrials.gov Identifier: NCT00565539). So far, there are no data available on the therapeutic potential of type III IFN administration in the context of IBD. An overview about the different IFNs and their respective role in GVHD and IBD pathogenesis can be obtained from **Table 1**.

**Table 1:** Overview about Type I, -II and -III IFNs and their role in GVHD and IBD pathogenesis.

	Type I IFN	Type II IFN	Type III IFN
**Members**	Mouse: α1, α2, α4-8, α11, α12-16, ϵ, κ, ζ	Mouse and human: γ	Mouse: λ2, λ3
	Human: α1, α2, α4-8, α10, α13, α14, α16, α17, α21, β, ϵ, κ, ω		Human: λ1-4
**Receptor expression**	Ubiquitously expressed on nucleated cells (195)	Ubiquitously expressed on nucleated cells (78)	Preferentially expressed on epithelial cells and some immune cells (e.g. DCs and neutrophils) (62, 80, 185, 196)
**IFN production**	In response to TLR3, RLR, cGAS and NOD1/2 stimulation (197–199)	In innate immunity: by NK- and NKT cells (75)	In response to TLR, RLR and Ku70 stimulation (200)
		In adaptive immunity: by CD4^+^ Th1 cells and CD8^+^ cells (63)	
**Effects in GVHD**	Positive modulation of murine disease outcome (121–125)	Detrimental effects of IFN-γ on murine intestinal epithelium (129–132)	In humans: SNPs in IFNL4 gene in donors of HSCT associated with increased risk of non-relapse mortality (146)
	Negative effects: increased GVHD and TRM occurrences after pre-transplant administration (120)	IFN-γ antagonism improved GVHD outcome (133)	
		Protective role *via* limiting the expansion of donor-derived T cells (134, 135) and donor-derived IL-12 in murine models (139, 142)	
		Several studies report evidence that IFN-γ regulates the alloreactive T cell pool and T cell expansion (134, 144)	
**Effects in IBD**	Protective effects (173–176)	Detrimental effects on murine intestinal epithelium (94)	Protective role in murine model of DSS-induced colitis (81, 126)
		Antiangiogenic effect on murine intestinal vasculature *in vitro* (181–183)	Increased levels in inflamed intestinal tissue and sera of CD patients (186)
		In murine DSS-colitis model: angiostatic activity in IBD and contributed to increased vascular permeability (179)	
		In humans: negative impact on intestinal barrier integrity (185)	

## Conclusion

To date, TNF is the sole proinflammatory cytokine that has been successfully targeted in IBD. Anti-TNF therapy with various anti-TNF antibodies (including infliximab, for example) is an essential backbone for the treatment of both CD and UC patients (201). Years of research and clinical success paved the way for increased interest in other cytokines and cytokine regulatory networks regarding the pathogenesis of IBD. Unfortunately, efforts in the field of anti-IFN therapy have not yet yielded promising results, as the use of fontolizumab, an anti-IFN-γ antibody, in CD patients did not result in improved disease outcome, and further investigation and development have been discontinued (189). With regard to the therapy of GVHD, IL-6 has been the best studied and targeted cytokine in this disease. Tocilizumab, an anti-IL-6 receptor antibody, has shown efficacy in steroid-refractory intestinal aGVHD as well as cGVHD (202, 203). IFN-γ in particular has been the focus of investigation in the context of GVHD. Due to divergent and pleiotropic effects of IFN-γ blockade in preclinical mouse models, no clinical studies have yet been conducted that consider direct targeting of IFN signaling pathways in intestinal GVHD.

Overall, it is clear that a highly complex and interconnected as well as -regulated cytokine network and its imbalance plays a crucial role in the process of mucosal intestinal inflammation as well as mucosal healing. Both non-hematopoietic and hematopoietic cells of the innate and adaptive immune systems each play a central role in disease pathogenesis. In the context of GVHD, a further complication is the need to distinguish between the effect of IFNs on donor cells and, on the other hand, on recipient cells, as underscored by various preclinical models. Other factors, such as different types of MHC-mismatched BMT mouse models or even the timing of treatment in the context of IFN-cytokine network therapy, must also be considered. Further research needs to be conducted to understand why and how IFNs play such pleiotropic roles in the development and progression of both IBD and GVHD. It would be desirable to investigate the presumably positive effect of type I interferons in IBD more closely to provide the basis for eventual clinical trials. In addition, the recently discovered type III IFNs still need to be characterized in more detail, as their receptors are preferentially expressed on epithelial cells. So far, not much is known about their presumed role in signaling networks in the field of intestinal homeostasis and inflammatory processes. Ultimately, it is critical to understand better the divergent downstream signaling cascades of IFNs, and how these are connected to inflammation or tissue protection. Separating these different effects and identifying targets downstream of IFNs or their receptors might prove a promising translational approach, as seen in the example of JAK inhibition. This knowledge is essential to pave the way for more effective clinical approaches by precisely addressing the expression or functions of IFNs in intestinal inflammation.

## Author Contributions

EH and PA developed the overall concept for this review article. EH collected and reviewed literature, discussed the studies, and wrote the first draft of the manuscript. PA and RZ critically revised the manuscript. All authors contributed to the article and approved the submitted version.

## Funding

PA is supported by the German Cancer Consortium (DKTK) (FR-01-375). The article processing charge was funded by the Baden-Wuerttemberg Ministry of Science, Research and Art and the University of Freiburg in the funding programme Open Access Publishing. RZ was supported by the SFB 1479 (P01) OncoEscape.

## Conflict of Interest

RZ received honoraria from Novartis, Incyte and Mallinckrodt.

The remaining authors declare that the research was conducted in the absence of any commercial or financial relationships that could be construed as a potential conflict of interest.
